# Pilot Study of Using Machine Learning to Detect Atherosclerotic Renal Artery Stenosis From Spectral Doppler Waveforms

**DOI:** 10.1016/j.ekir.2025.01.012

**Published:** 2025-01-16

**Authors:** Haseeb Mukhtar, Seyed Moein Rassoulinejad-Mousavi, Shahriar Faghani, Bradley J. Erickson, Sanjay Misra

**Affiliations:** 1 Department of Radiology, Mayo Clinic, Rochester, Minnesota, USA; 2 Vascular Interventional Radiology Translational Laboratory, Mayo Clinic, Rochester, Minnesota, USA; 3 Radiology Informatics Laboratory, Mayo Clinic, Rochester, Minnesota, USA; 4 Mayo Clinic Platform, Mayo Clinic, Rochester, Minnesota, USA; 5 Division of Nephrology and Hypertension, Mayo Clinic, Rochester, Minnesota, USA

**Keywords:** atherosclerosis, Doppler, duplex scan, hypertension, machine learning, renal artery stenosis

## Abstract

**Introduction:**

We investigated whether machine learning (ML) could be used to determine atherosclerotic renal artery stenosis (ARAS) using spectral Doppler waveforms in renal duplex ultrasound (DUS).

**Methods:**

Patients with unilateral ARAS (contralateral normal kidney) confirmed by angiogram and requiring renal artery stent placement were retrospectively identified from January 2000 to January 2022. The exclusion criteria were unavailable preoperative renal DUS images, concomitant fibromuscular dysplasia, more than 1 renal artery on either side, or a previously placed renal artery stent with in-stent restenosis. Two hundred patients were selected; the affected kidney was used as the positive case and the contralateral kidney was used as the control. The spectral waveforms were reconstructed by manually tracing the outer envelope using WebPlot Digitizer. The graphical coordinates were then converted into 1-dimensional velocity signals. Signals were labeled as ARAS and normal and then randomly divided into training (80%) and testing (20%) datasets. A 1-dimensional convolutional neural network (CNN) was trained to classify the signals and detect ARAS. An Adam optimizer with a learning rate of 0.001 and a cross-entropy loss function were utilized. Five-fold cross-validation was applied, and the model was trained for 1000 epochs.

**Results:**

A total of 396 signals were used from 198 patients after excluding 2 patients because of inadequate signal extraction (median age = 72 years, females = 51.0%). The overall accuracy of the trained model was 0.95 with a precision of 0.94. The area under the receiver operating characteristic curve was 0.97.

**Conclusion:**

ML has been successfully employed to detect ARAS using arterial spectral Doppler waveforms in DUS.

## Graphical abstract

**Figure:**
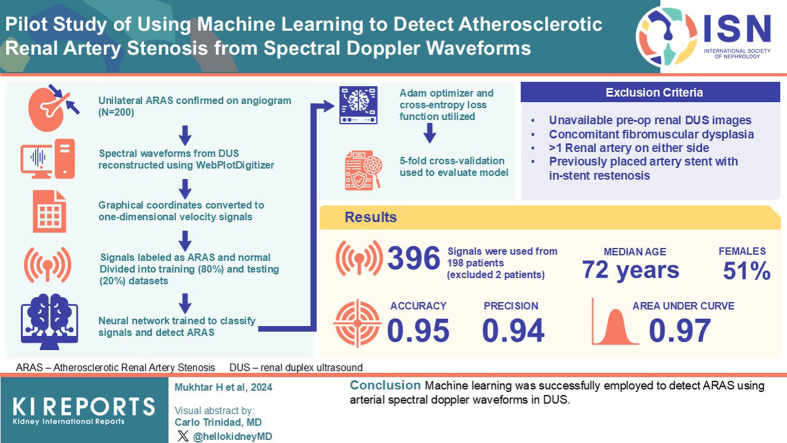

Renal artery stenosis (RAS) is the narrowing of the renal artery, which impairs blood flow to the kidneys. It is one of the leading causes of secondary hypertension and compromised renal function.^1^ ARAS is the most common subtype (90%) and is caused because of the deposition of atherosclerotic plaques around the vessel.^2^^,^^3^ ARAS is especially prevalent among individuals aged > 65 years (7%) and those with diabetes and secondary hypertension (25%).^4^ Therefore, with increasing life expectancy and prevalence of cardiovascular risk factors, ARAS prevalence is expected to rise further.

There are many diagnostic modalities used to detect ARAS, among which one of the most common is ultrasonography (US).^5^^,^^6^ Renal DUS is a specialized type of US that combines anatomical and flow US to provide meaningful insights of the kidney and its vasculature. It is preferred because of its noninvasiveness, time efficiency, safety, and low cost.^1^^,^^7^ Hemodynamically significant RAS is defined as vessel narrowing of ≥ 70% by visual estimation or 50% to 69% narrowing, together with 1 of the following: (i) a resting or hyperemic systolic translesional gradient of ≥ 20 mm Hg, (ii) a mean gradient ≥ 10 mm Hg, (iii) hyperemic gradients of the same magnitude, or (iv) a fractional flow reserve of < 0.8.^8^ The frequently used parameters to assess significant RAS are peak systolic velocity, renal-aortic ratio (i.e., ratio of the peak blood flow velocities in the renal artery to the aorta), lack of Doppler US signal in cases of occlusion, and the tardus-parvus waveform, among others.^9^ DUS has proven to be a powerful initial diagnostic tool with sensitivity going as high as 91% with a peak systolic velocity ≥ 180 cm/s. Similarly, the presence of a tardus-parvus waveform distal to the stenosis yielded a specificity of 96% and a positive predictive value of 92%.^10^^,^^11^

ML has shown immense potential in diagnostic and interventional radiology.^12^^,^^13^ Studies have investigated the application of ML to interpret Doppler waveforms. Models have been able to diagnose and predict prognoses based on echocardiograms and peripheral arterial Doppler signals.^14^, ^15^, ^16^, ^17^

In this study, we sought to determine whether ML can be applied to renal DUS waveforms to identify clinically significant RASs that warrant interventional treatment and stent placement.

## Methods

### Patient Selection

This retrospective study was conducted after obtaining approval from the institutional review board (approval protocol number: 21-010671) of Mayo Clinic. The study was deemed minimally invasive, and informed consent from the patients was waived. Our study included patients with unilateral ARAS and a contralateral normally functioning kidney who underwent stent placement in the stenotic vessel. ARAS was confirmed using renal angiography during the intervention. Angiograms are still considered the gold standard for RAS diagnosis.^1^ Patients were excluded if preoperative duplex scan images were unavailable, they had > 1 renal artery on either side, a previously placed renal artery stent with in-stent restenosis, or RAS because of nonatherosclerotic causes such as fibromuscular dysplasia. Patients were identified between January 2000 and January 2022, and 200 patients were randomly selected from this cohort. This was done to account for changes in the radiological practice of renal duplex scans over 22 years. Demographic and imaging data were obtained from each patient after a detailed chart review. Descriptive analysis was performed using R software v4.3.2 (R Foundation for Statistical Computing).

### Ultrasound Machines and Technique

Renal DUS was performed by radiologists at our institution using ultrasound machines from different manufacturers (Siemens Healthineers, Erlangen, Germany; GE Healthcare, Chicago, IL; and Philips Medical Systems, Amsterdam, Netherlands). The examination began with identification of the kidneys and calculation of the renal length (the longest distance between the upper and lower poles of the kidney), followed by color Doppler analysis beginning from the abdominal aorta and progressing distally along the renal arteries. The angle of the transducer relative to the blood flow was kept at ≤ 60°.

### Envelope Extraction and Data Set Preparation

**Figure 1:**
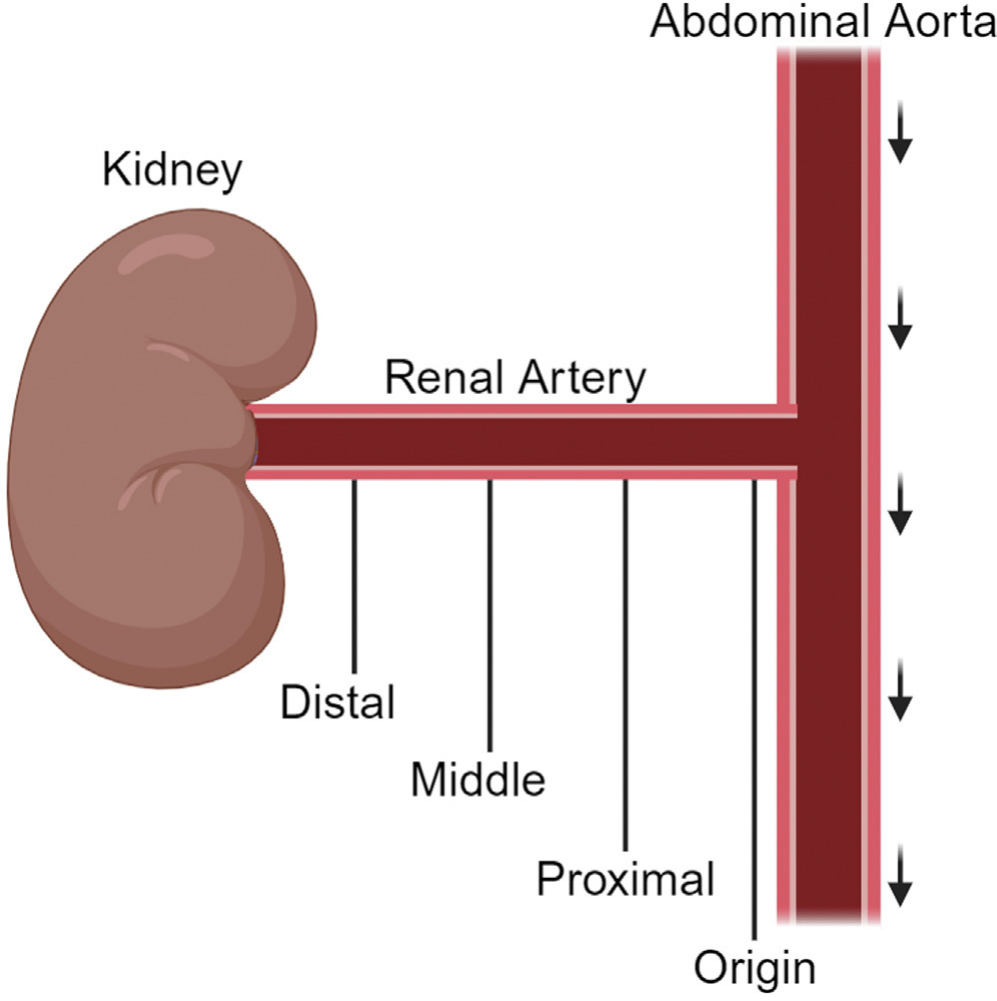
Divisions of the renal artery.

Preoperative renal duplex scans were reviewed*, and images showing the ARAS site* were identified and saved. One image per patient was selected*, and the site was noted. The site of* RAS *is mentioned in the duplex scan image with respect to its distance from the abdominal aorta as the origin, proximal, middle, or distal (*Figure 1). Duplex images from the contralateral normal kidney were also reviewed*, and* 1 *representative image from the corresponding site was selected as the control (i.e., if the site of stenosis was in the proximal renal artery,* 1 image was selected from the proximal renal artery of the normal kidney). The purpose of selecting control images from the same patient was to account for interoperator and interpatient variability.

**Figure 2:**
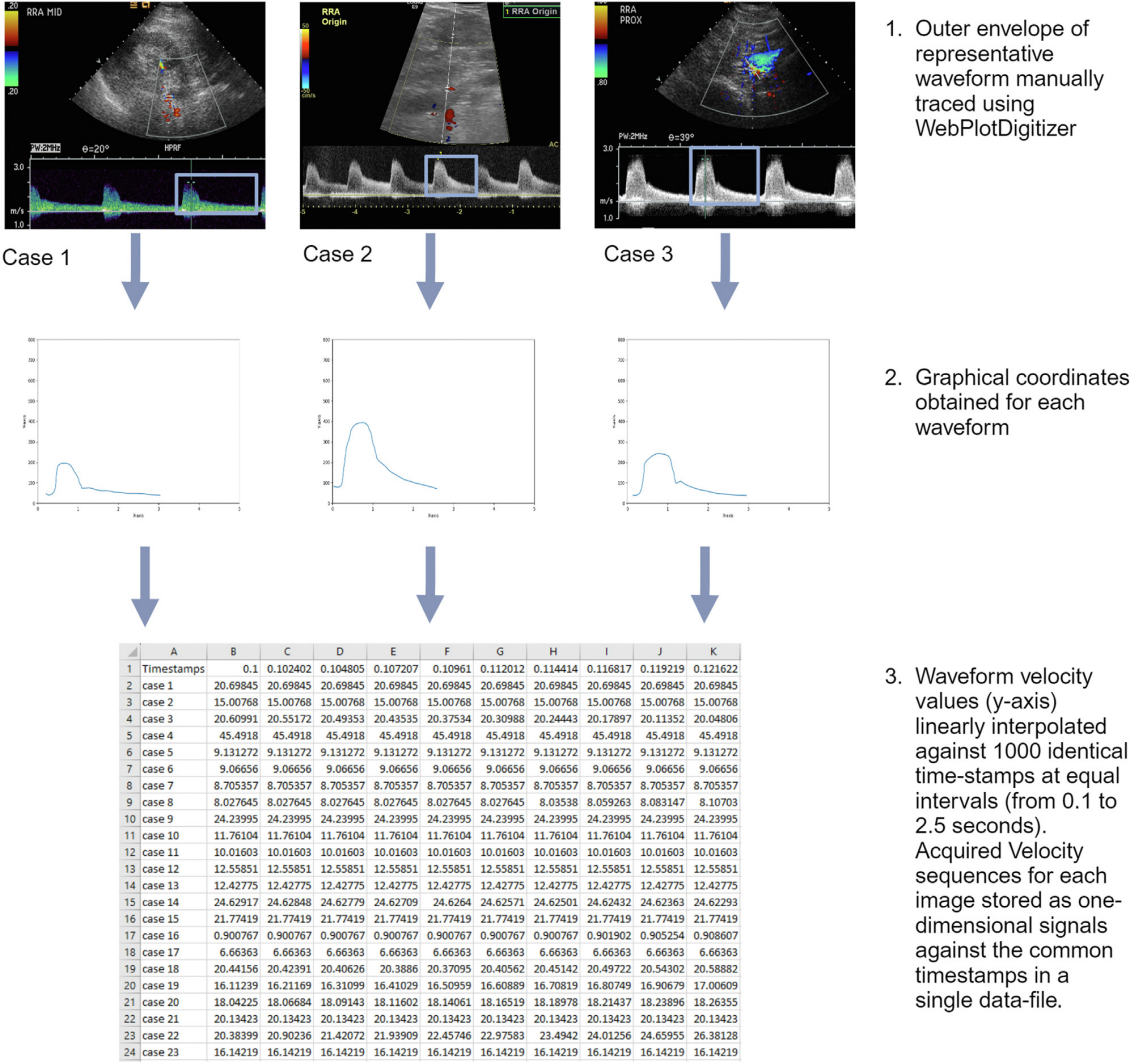
Spectral waveform manual reconstruction and signal extraction.

**Figure 3:**
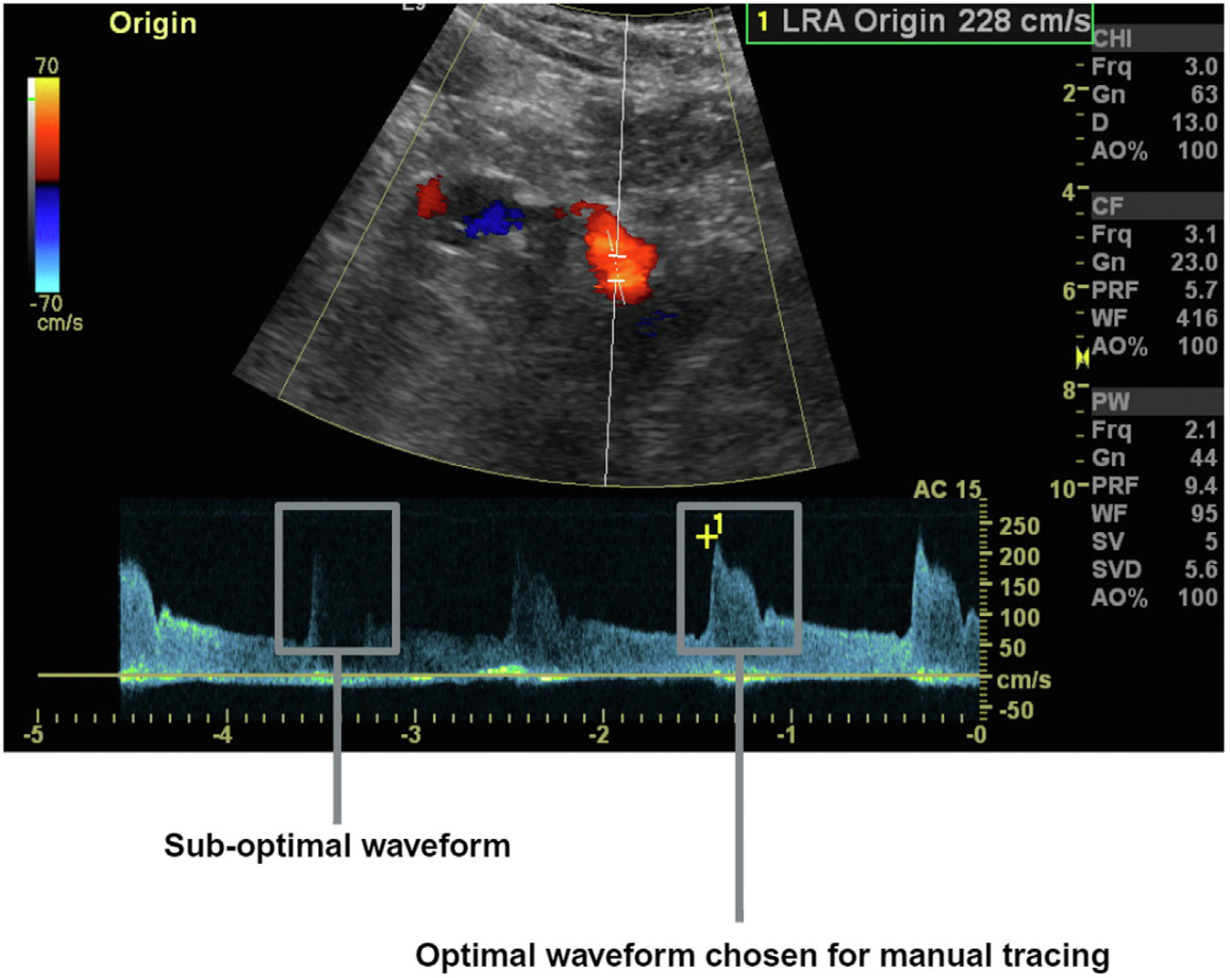
An example of suboptimal waveforms encountered.

*In*Figure 2, we illustrate how the Doppler signal was extracted from the renal duplex scans. Each duplex image was entered into an open-source software program called WebPlotDigitizer (version 4.6).^18^ This allowed digital graphical plots to be obtained from the images. Scale calibration was first performed, followed by manual tracing of the outer envelope on a computer. Because there were some duplex images with inadequate or distorted waveforms (Figure 3), the coordinates of only 1 representative waveform were extracted from each image. Tracing began immediately before the systolic upstroke and ended before the next upstroke. This method of tracing and the acquired individual plots were agreed upon by all the authors before proceeding to the next step. To make the time stamps identical, the velocity values were interpolated linearly from 0.1 to 2.5 s against 1000 timestamps at equal intervals. One thousand timestamps were chosen to obtain smooth resultant waveforms that were as similar as possible to the original waveforms. Subsequently, a datasheet of 1-dimensional velocity values against the common timestamps was prepared each for the 2 classes of signals (ARAS [class 1] and control [class 2]).

### Model Used

In this study, we aimed to train and assess the performance of a CNN model for classifying 2 groups of signals: ARAS and control. Using 1-dimensional velocity signals as the input, the goal was to evaluate how well the model could differentiate between these 2 classes and to assess the consistency of its predictions across multiple folds of cross-validation.

### Data Processing and Splitting

The data, comprising of 1-dimensional velocity signals for the 2 classes, were combined into a single data set, and the corresponding class labels were assigned. We then split the data set into training and test sets using a traditional 80/20 split, where 80% of the data were used for training and validation, and 20% were used for final testing.

Instead of traditional limited data splits, we opted for a 5-fold cross-validation approach using the entire data set for training within each fold. This decision allows the model to leverage all available data, improve its ability to learn meaningful patterns, and make the results more robust.

### Cross-Validation Process

Five-fold cross-validation was used to evaluate the performance of the model. In each fold, the data set was divided into 5 parts, with 1 part used for validation, and the remaining 4 parts used for training. This process was repeated 5 times, using a different fold for validation and the rest for training. Thus, the model was tested on all parts of the data set, ensuring that every data point was used for both training and validation, thus providing a more reliable estimate of the model's performance.

In addition, to investigate the consistency of the predictions, we tracked whether the model predicted the same class for individual patients across different folds. This study aimed to assess whether the decisions of the models were stable and reproducible.

### Model Architecture

CNNs are well-suited for extracting hierarchical features from structured input data. We used a 1-dimensional CNN (1-D CNN) because the input to the network was a 1-dimensional velocity signal for each patient, which was trained to predict whether the patient belonged to the ARAS or healthy group.

**Figure 4:**
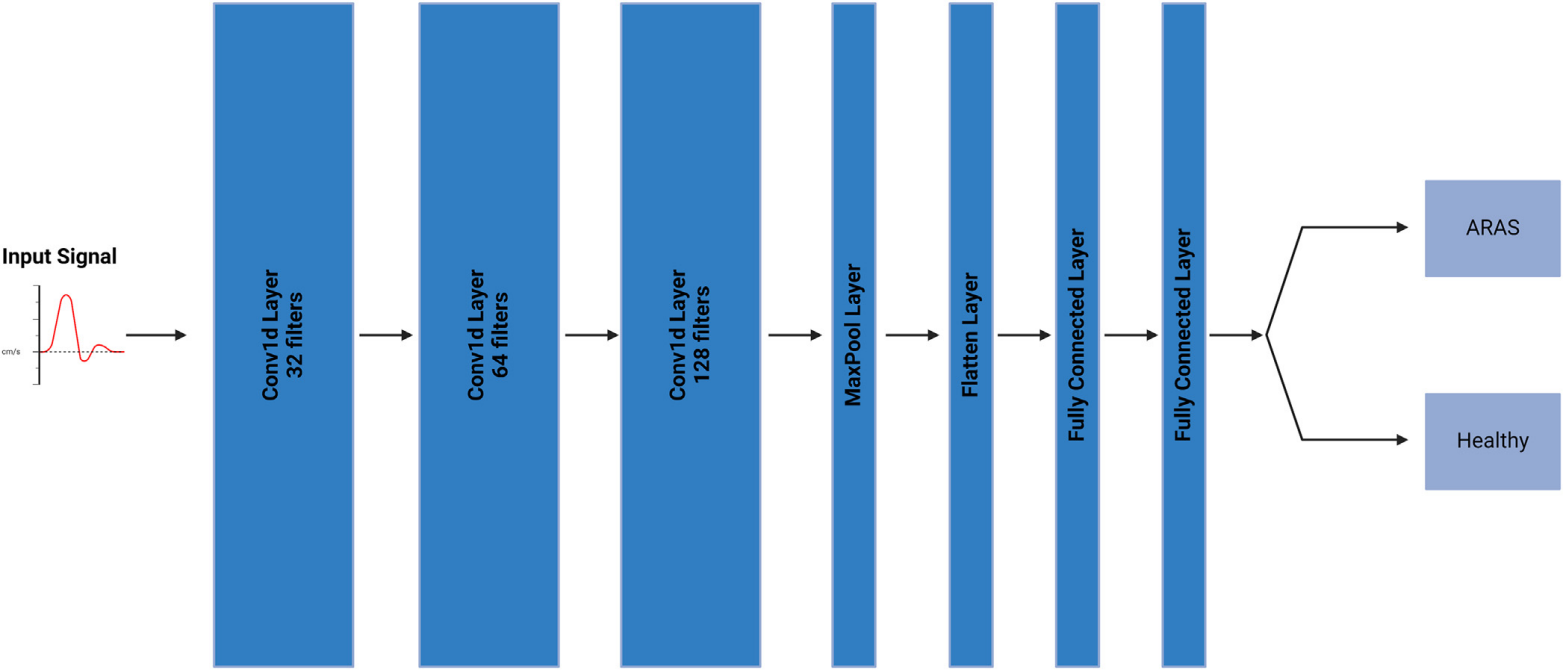
Model architecture. ARAS, atherosclerotic renal artery stenosis; ReLU, Rectified Linear Unit.

The CNN consists of 3 convolutional layers, each followed by maximum pooling to reduce dimensionality. The first convolutional layer had 32 output channels, the second layer increased the number to 64, and the third layer had 128 channels. After the convolutional layers, the feature maps were flattened and passed through 2 fully connected layers: one hidden layer with 128 neurons and an output layer with 2 neurons corresponding to the 2 classes. The softmax activation function was used in the output layer to obtain the probabilities for each class (Figure 4).

The model was trained for 500 epochs with a batch size of 32 using the Adam optimizer with a learning rate of 0.001 and a cross-entropy loss function, which is the standard for classification tasks. Cross-entropy is a widely used loss function that measures the performance of ML classification models. It calculates the degree of error in the model’s predictions, and this is then used as a guide during the optimization phase.^19^ Adaptive Moment Estimation is an extension of the stochastic gradient descent algorithm, commonly used in ML to optimize small to moderately sized datasets by adjusting learning parameters and improving model accuracy ^20^

## Results

### Patient Demographics

As mentioned above, our final data set comprised 198 patients, with 2 signals (1 ARAS and 1 control) extracted from each patient. In Table 1, we summarize the demographic information of the patients. The median age of the patients at the time of the duplex scan was 72 (range: 43–89) years.Of the total participants, 51.0% were female. The median estimated glomerular filtration rate, creatinine level, and blood urea nitrogen level were 41.0 (range: 8.1–138.0) ml/min per 1.73 m^2^, 1.50 (range: 0.5–5.6) mg/dl, and 39.5 (range: 16.0–85.0) mg/dl, respectively.

**Table 1:** Patient demographics

Sex	
Male	97 (49.0%)
Female	101 (51.0%)
Age, yrs^a^	72 (43–89)
Side of stenotic renal artery	
Left	105 (53.0%)
Right	93 (47.0%)
BUN (mg/dl)^a^	39.5 (16.0–85.0)
Serum creatinine (mg/dl)^a^	1.50 (0.5–5.6)
eGFR (ml/min per 1.73 m^2^)^a^	41.0 (8.1–138.0)
Smoker	
Current	32 (18.1%)
Former	96 (54.2%)
Never	49 (27.7%)
Hypertension	
Yes	192 (98.0%)
No	4 (2.0%)
Hyperlipidemia/dyslipidemia	
Yes	137 (70.3%)
No	58 (29.7%)
CKD	
Yes	185 (98.4%)
No	3 (1.6%)
Known CKD stage	
Stage 1/2/3a	72 (40.7%)
Stage 3b/4/5	105 (59.3%)
Coronary artery disease	
Yes	107 (54.6%)
No	89 (45.4%)
Diabetes mellitus	
Yes	60 (30.8%)
No	135 (69.2%)

BUN, blood urea nitrogen; CKD, eGFR, estimated glomerular filtration rate; TIA, transient ischemic attack.

Categorical variables are reported as counts with percentages in parenthesis (%)

a These are continuous variables and are reported as median with range in parenthesis.

### Model Performance

The results of this study demonstrate the performance and reliability of a CNN model for classifying patients into 2 groups, ARAS and healthy, based on 1-dimensional velocity signals. To ensure a robust evaluation, we employed a 5-fold cross-validation approach utilizing the entire data set for training each fold. This method provides a comprehensive assessment of the ability of a model to be generalized across different subsets of data. In addition to evaluating the model's overall performance, we investigated the consistency of its predictions across folds, specifically assessing whether the model consistently classified individual patients in the same manner across different training-validation splits. The following sections outline the key metrics obtained from the cross-validation process and highlight the stability and effectiveness of the model in this classification task.

### Cross-Validation Metrics

The results of the 5-fold cross-validation along with the computed statistics for each metric, provide a detailed view of the model's performance across different subsets of the data. The metrics assessed include accuracy, precision, recall, F1 score, and the area under the receiver operating characteristic curve. The following is an analysis of these findings. The calculated statistics (mean, SD, variance, and 95% confidence interval [CI]) for each metric are presented in Table 2.

**Table 2:** Results of 5-fold cross validation

Metric	Mean	SD	Variance	95% CI
Accuracy	0.9416	0.0205	0.0004	0.9237–0.9596
Precision	0.9487	0.0568	0.0032	0.8989–0.9985
Recall	0.9280	0.0353	0.0012	0.8970–0.9589
F1 score	0.9368	0.0318	0.0010	0.9090–0.9647
ROC AUC	0.9759	0.0159	0.0003	0.9620–0.9899

CI, confidence interval; ROC AUC, area under the receiver operating characteristic curve.

In Table 2, we show that the mean accuracy across the 5 folds was 0.9416, indicating that the model correctly classified approximately 94% of the samples on average. An SD of 0.0205 indicated modest variation across the folds, reflecting a stable classification performance. This is further supported by a variance of 0.0004, which confirms low variability. With a 95% CI ranging from 0.9237 to 0.9596, the true accuracy is likely to fall within this range across the different datasets.

For precision, the model achieved a mean value of 0.9487, demonstrating a high proportion of true-positive predictions among all positive predictions. This highlights the capability of the model to effectively minimize false positives. An SD of 0.0568 and a variance of 0.0032 suggested moderate variation across folds, whereas the 95% CI (0.8989–0.9985) indicated that the true precision was expected to lie within this range, reflecting stable performance.

The recall results showed a mean of 0.9280, indicating that the model identified 92.8% of the true-positive samples. An SD of 0.0353 indicates some variability across folds, with a variance of 0.0012, offering additional confirmation of this observation. The 95% CI (0.8970–0.9589) provides a range in which true recall likely resides, underscoring the robust performance across datasets. The small SD and variance, as well as the narrow CI and high mean recall, indicate consistent and reliable performance overall and eliminate concerns about minor variabilities.

In terms of the F1 score, a mean value of 0.9368 represented a balance between precision and recall, with the model performing consistently in minimizing false positives and maximizing true positives. An SD of 0.0318 and variance of 0.0010 indicated low variability across folds. The 95% CI (0.9090–0.9647) suggests the true F1 score is expected to fall within this range, highlighting a reliable performance.

The area under the receiver operating characteristic curve score averaged 0.9759, indicating the strong ability of the model to differentiate between positive and negative classes. An SD of 0.0159 and variance of 0.0003 reinforce the consistency of this metric across the folds. The 95% CI (0.9620–0.9899) further underscores the robust capacity of the model for classification.

Across all the metrics, the narrow CIs demonstrated consistent and reliable performance across the validation folds. These intervals suggest that the actual performance of the full data set is likely to align closely with the observed results. This consistency indicates a well-balanced model showing no signs of overfitting or underfitting, as evidenced by the relatively low variation in the results.

### Consistency of Predictions for Each Signal

An important aspect that we wanted to investigate as an extra assurance check was the consistency of the predictions across different folds for each signal. To assess the stability of model predictions, we tracked whether each signal received the same classification (ARAS or control) for each fold. The results showed that the model was consistent in its predictions across all folds, with no patient receiving different predictions. This is a promising sign, because it indicates that the model's decisions are stable and do not produce conflicting results for the same signal when trained on different data subsets.

### Final Model Performance

**Figure 5:**
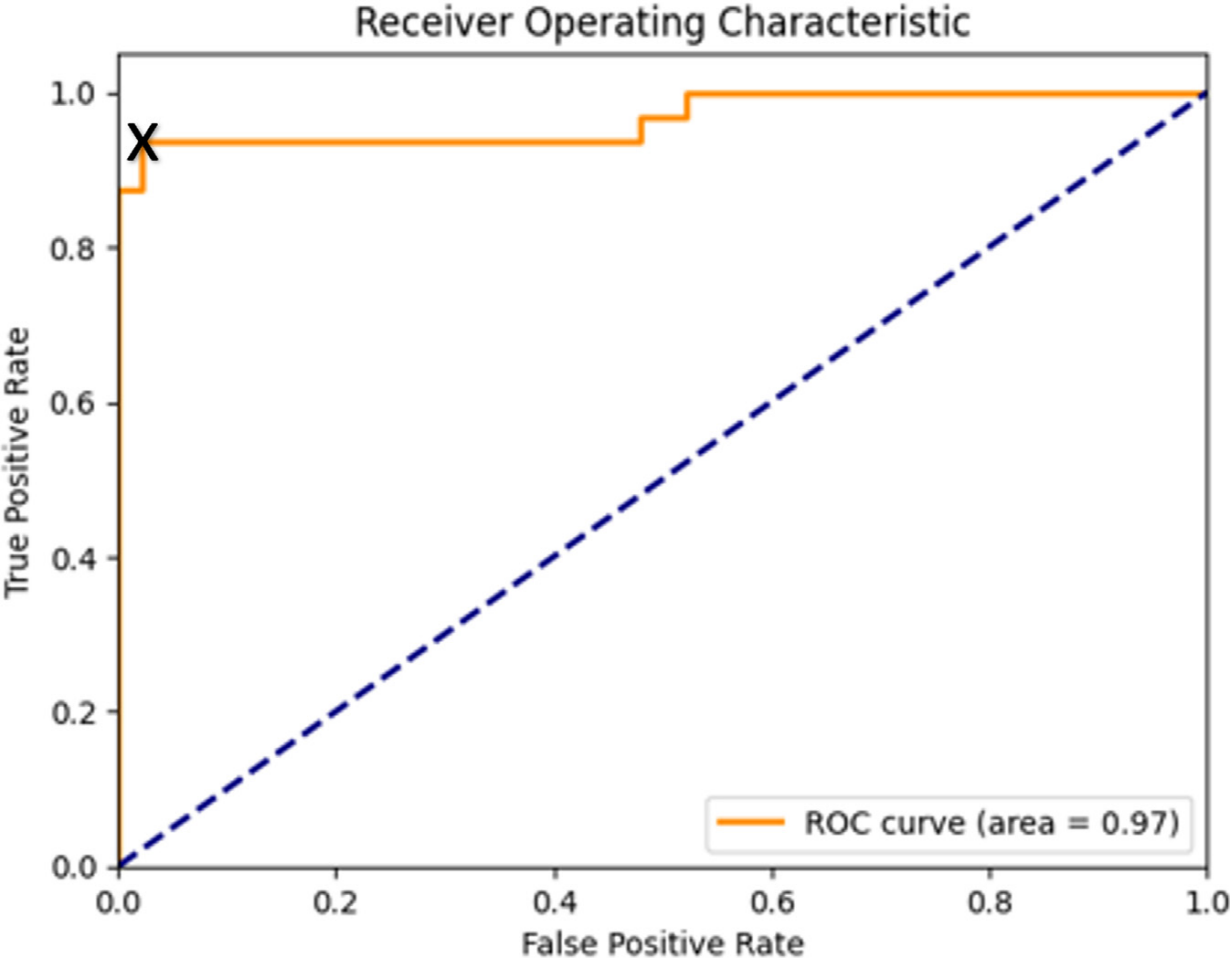
*Receiver Operator Characteristic (ROC) curve.* Operating point for model marked (X).

**Figure 6:**
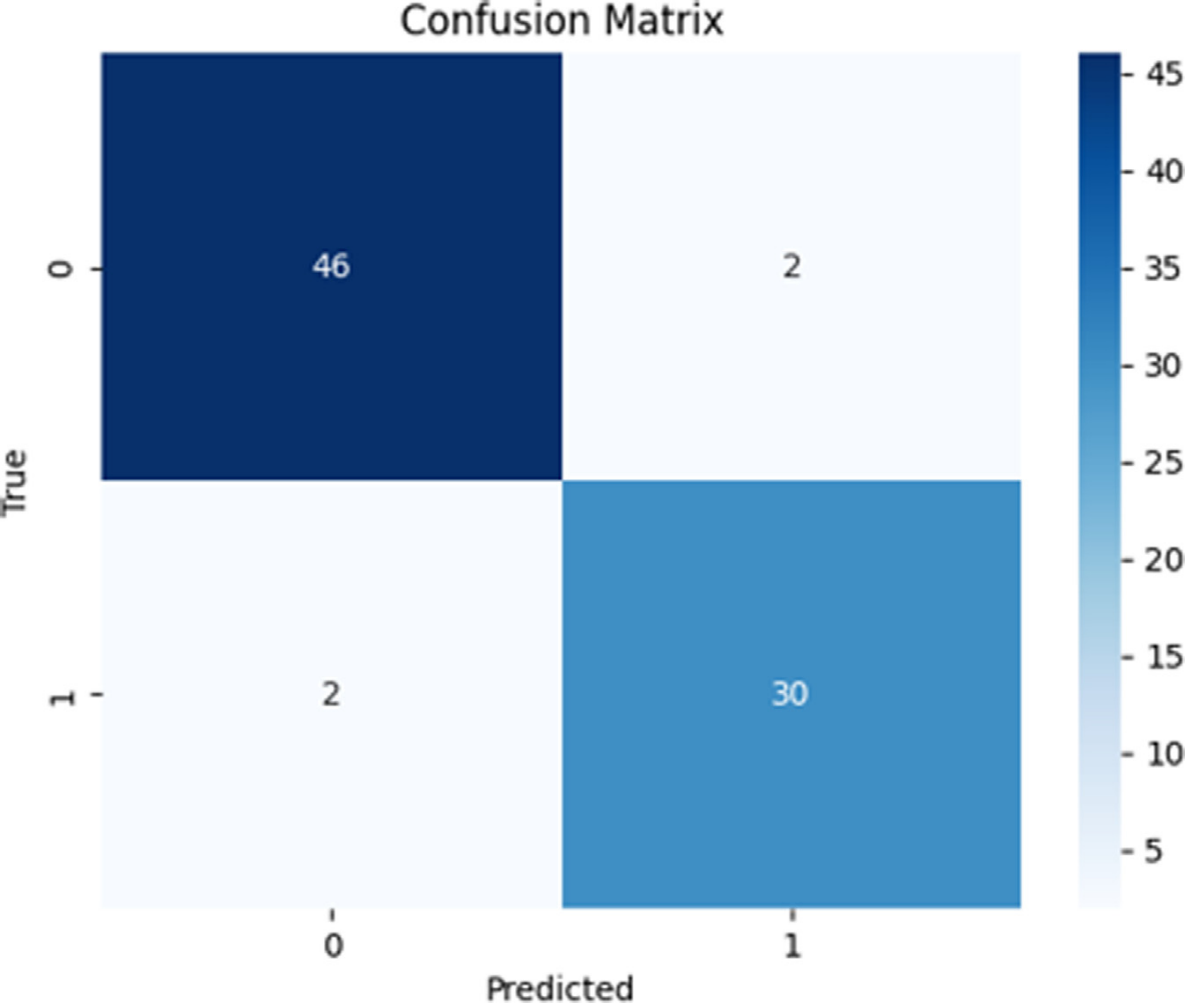
Confusion matrix of the model prediction on the unseen test data set.

After cross-validation, the model was retrained on the entire data set, and the final evaluation metrics were calculated using the test set. In Table 3, we present the overall model after the inference of the unseen test set. The model was able to discriminate between the ARAS and control signals with an accuracy of 0.95, a specificity of 0.96, and a precision of 0.94. The area under the receiver operating characteristic curve was 0.97 as shown in Figure 5. In Figure 6, we illustrate the confusion matrix of the model.

**Table 3:** Performance of the final model

Accuracy	0.95
Specificity	0.96
Precision	0.94
Recall	0.94
F1 score	0.94
ROC AUC	0.97

ROC AUC, area under the receiver operating characteristic curve.

## Discussion

ARAS typically presents in older patients with hypertension or renal impairment and has a similar prevalence in either sex - males and females.^21^ The current patient cohort had an almost equal sex distribution (51.0% vs 49.0%), a median age of 72, and a prevalent presentation of hypertension (98.0%) and chronic kidney disease (98.4%). Thus, it adequately represented the population affected by ARAS.

Severe ARAS leads to various complications including secondary hypertension, pulmonary edema, left ventricular dysfunction, cerebrovascular disease, ischemic nephropathy, and renal failure.^22^ Timely intervention can avoid irreversible sequelae secondary to the renal damage that occurs.^4^ Therefore, clinically significant RAS must be picked up early and treated accordingly. Ultrasound is the preferred initial diagnostic modality because of its favorable characteristics. However, its user-dependent nature and learning curve make it challenging, especially for those with less training.^14^^,^^23^ The objective nature of artificial intelligence (AI) overcomes the shortcomings of US, as discussed by De Jesus-Rodriguez *et al.*^24^ No doubt, with proper implementation, AI can improve clinical workflow and further increase reliability and accessibility for kidney US. This pilot study highlights the significance of spectral Doppler and its applicability in training an AI model for timely detection of significant vessel stenoses. By incorporating such AI models into the DUS machine software, renal artery stenoses can be identified more efficiently, and the respective patients can be identified more promptly.

In Table 4, we list the previous studies that trained AI models on arterial Doppler waveforms. Very limited data are available on the application of AI in the diagnosis of RAS. To the best of our knowledge, another study has employed AI to detect RAS.^25^ This work demonstrated the highest accuracy of 83.49% (on the test data set) using multimodal data as opposed to solely spectral waveform data (accuracy of 81.18% on the test data set). The highest-performing model was based on the ResNeSt architecture.^25^

**Table 4:** Previous AI models used for Doppler signal interpretation

	Study	Radiological modality	Data set	Model architecture	Model performance	Reference
1	Detecting peripheral artery disease (PAD) in background of diabetes	Duplex ultrasound	Total = 590 PAD = 369 No PAD = 221	Logistic regression (multiscale wavelet variance features and statistical features)	Accuracy = 88% AUC = 0.93	Normahani *et al.*^14^
2	Detecting PAD and predicting ankle-brachial index (ABI)	Arterial Doppler	Total = 3432 PAD = 1941 No PAD = 1491	InceptionTime	Accuracy = 85% AUC = 0.94	McBane *et al.*^15^
3	Diagnosing RAS using deep learning and multimodal fusion	Multimodal data	RAS = 449 patients Without RAS = 1036	ResNeSt, ResNet, XCiT	Highest model accuracy = 92.26% on validation data set and 83.49% on test data set	Wang *et al.*^25^
4	Predicting major adverse outcomes among patients with PAD	Arterial Doppler	Total = 10437	InceptionTime	AI analysis significantly associated with all-cause death at 1 year (HR=1.66), MACE (HR=2.12) and MALE (HR=9.23)	McBane *et al.*^26^
5	Diagnosing atherosclerosis from carotid artery Doppler signals	Arterial Doppler	114 subjects (60 patients, 54 healthy volunteer)	Artificial Immune System	Accuracy = 99.33%	Latifoglu *et al.*^16^

AI, artificial intelligence; AUC, area under the curve; HR, hazard ratio; MACE, major adverse cardiac event; MALE, major adverse limb events; PAD, peripheral artery disease; RAS, renal artery stenosis.

As mentioned above, significant work has been conducted on the application of ML to Doppler waveforms for diagnosis and/or prognosis, particularly in cardiology and peripheral artery disease. Normahani *et al.*’s ^14^ study is the most similar to ours. They used DUS of the anterior and posterior tibial arteries to detect peripheral artery disease. They manually reconstructed the outer envelope of the spectral waveform in a similar manner using the R software package Digitize (version 0.0.4). Subsequently, a long short-term memory network was trained on the extracted raw signals. In addition, logistic regression and support vector machine models were trained on the features extracted from the raw signals. The highest accuracy achieved was 88% using a logistic regression model trained on statistical and wavelet transform features.^14^

McBane *et al.*^15^ trained a model to detect peripheral artery disease using posterior tibial artery waveforms. However, this study used only Doppler ultrasound (instead of DUS images, as in our case). Their model was based on the InceptionTime architecture and achieved an accuracy of 89% on a data set of 3432 patients. A different study done by similar authors again utilized arterial Doppler ultrasounds to predict major adverse cardiac and major adverse limb events among patients with peripheral artery disease. This model was based on the InceptionTime architecture and was trained on a data set of 10,437 patients. The model successfully predicted all-cause death at 1-year, major adverse cardiac events, and major adverse limb events with hazard ratios of 1.66, 2.12, and 9.23, respectively.^26^

Our model architecture is a 1-D CNN, which performs remarkably well on sequential time-series data.^27^ Lin *et al.*^28^ employed a 1-D CNN to successfully assess arteriovenous shunt stenosis using phonoangiographic signals. They achieved an accuracy of 86.82%. In comparison with these other studies, the current model performed well with an accuracy of 90.41%. Interestingly, the model was trained solely on the extracted velocity signals, as opposed to the extracted features or multimodal data, as in the aforementioned studies. We employed 5-fold cross-validation during model development to address concerns regarding overfitting. The results across the 5-fold treatments were consistent according to the statistical analysis, as shown in Table 2. The consistency of predictions across folds indicates that the model's decisions are stable and reproducible, which is crucial in clinical settings, where consistency is paramount.

One thing to note is that our classifier model specifically detected clinically significant stenosis that underwent stent placement. Scenarios that warrant stent placement include sudden onset flash pulmonary edema, accelerating decline in renal function, and failure to control hypertension on 3 maximally tolerated medications (including 1 diuretic).^8^ The model is more suitable for determining stenoses that require interventional treatment as opposed to those that are managed solely on medical therapy.

This study had some limitations. Manual tracing of the outer envelope makes the signal digitalization process exhaustive and inevitably imperfect. In addition, the author performing the manual tracing was not blinded to whether the waveform indicated significant stenosis or control thereby bringing in the risk of subjectivity and bias. Future attempts at automating this process of outer envelope extraction will not only make it quicker and more efficient, but also less subjective thereby eliminating any need for blinding for the sake of increasing reliability. In a pilot study, our model was trained and tested using a single-center retrospective data set. The limited data set brings with it concerns of inadequate training and model overfitting. Although 5-fold cross-validation was used to tackle this concern, future projects may improve this by using larger datasets and incorporating external validation to further assess model generalizability. This could be achieved by working with larger patient cohorts and prospective multicenter datasets. As mentioned earlier, the data set comprised patients who were referred for renal artery stent placement; taking the opposite side as the control resulted in a case proportion of 50%, which may not align with the real-world case distribution. Future studies may attempt to include, in the data set, patients that underwent DUS followed by angiogram with no RAS identified as controls. Finally, clinically significant stenosis ranged from 50% to >70% narrowing.^8^ Our model was not able to determine the exact degree of stenosis, which can be a useful parameter when managing ARAS.

In conclusion, a 1-D CNN model was successfully trained solely on the spectral arterial Doppler waveforms to detect ARAS. This study highlights the applicability of 1-D CNN models to such tasks and could potentially open the door to automating the diagnosis and management of clinically significant ARAS. Future directions of this study include developing a model that can accurately determine the degree of stenosis and utilizing AI techniques to predict the risk of in-stent restenosis or progression to renal replacement therapy after renal artery stenting.

## Disclosure

SM declared the following: received grants from NIH for projects R01 HL098967 and DK135407; licensed patents to Pavaj Vascular, Medtronic, Penumbra, Humacyte, American Heart Association; Founder of Pavaj Vascular; and equity in Inova Vascular. All the other authors declared no competing interests.

## Supplementary Material

Supplementary File (PDF) TRIPOD Checklist.
